# The effects of tibial fracture and Ilizarov osteosynthesis on the structural reorganization of sciatic and tibial nerves during the bone consolidation phase and after fixator removal

**DOI:** 10.1007/s11751-015-0227-1

**Published:** 2015-08-08

**Authors:** Tatyana N. Varsegova, Natalia A. Shchudlo, Mikhail M. Shchudlo, Marat S. Saifutdinov, Mikhail A. Stepanov

**Affiliations:** Russian Ilizarov Scientific Center for Restorative Traumatology and Orthopaedics, 6, M.Ulyanova Street, Kurgan, Russian Federation 6640014

**Keywords:** Shin bone fractures, Nerve fibres degeneration, Dogs

## Abstract

Reactive and adaptive changes in mechanically uninjured nerves during fracture healing have not been studied previously although the status of innervation is important for bone union and functional recovery. This study explores whether subclinical nerve fibre degeneration occurs in mechanically uninjured nerves in an animal fracture model and to quantify its extent and functional significance. Twenty-four dogs were deeply anaesthetized and subjected to experimental tibial shaft fracture and Ilizarov osteosynthesis. Before fracture and during the experiment, electromyography was performed. In 7, 14, 20, 35–37 and 50 days of fixation and 30, 60–90 and 120 days after fixator removal, the dogs were euthanized. Samples from sciatic, peroneal and tibial nerves were processed for semithin section histology and morphometry. On the 37th postoperative day, M-response amplitudes in leg muscles were 70 % lower than preoperative ones. After fixator removal, these increased but were not restored to normal values. There were no signs of nerve injuries from bone fragments or wires from the fixator. The incidence of degenerated myelin fibres (MFs) was less than 12 %. Reorganization of Remak bundles (Group C nerve fibres—principally sensory) led to a temporal increase in numerical nerve fibre densities. Besides axonal atrophy, the peroneal nerve was characterized with demyelination–remyelination, while tibial nerve with hypermyelination. There were changes in endoneural vessel densities. In spite of minor acute MF degeneration, sustained axonal atrophy, dismyelination and retrograde changes did not resolve until 120 days after fracture healing. Correlations of morphometric parameters of degenerated MF with M-response amplitudes from electromyography underlie the subclinical neurologic changes in functional outcomes after tibial fractures even when nerves are mechanically uninjured.

## Introduction

The shaft of the tibia is the commonest site of closed and open fractures, but the optimum treatment option remains the subject of debate. The standard treatment for tibial diaphyseal fractures is intramedullary nailing. This treatment option has resulted in a good ability for the patients to return to work, especially after interlocked nailing [1]. In some cohort studies, a high rate of complications from intramedullary nailing has been described [2, 3]. Even in series with a low rate of complications after intramedullary nailing, 60 % of patients experienced limitations in activity and restrictions in quality of life and 44 % reported knee pain [4].

An alternative, external fixation and the Ilizarov fixator, has been labelled “a panacea for the poor” [5] and has found wide use in developing countries [6]. Ilizarov osteosynthesis is considered as a preferred, safe and effective method in open, wedged and complex tibial fractures [7–11], but one disadvantage is the use of wires situated close to nerves [12]. Theoretically, disorders of nerves of the central or peripheral system can have substantial influence on bone health and repair [13], but little is known about reorganization of nerves after fractures in the extremities [14]. The well-known classification of nerve injuries are applied usually to clinical evident cases of nerve injury [15], but the role of peripheral nerves in posttraumatic skeletal pain, functional recovery and bone healing is poorly understood [16], although in tissue samples of aseptic delayed union or nonunion of diaphyseal bones the paucity or total lack of peripheral innervation was marked [17].

## Aim

The aim of the study was to identify destructive and adaptive histological changes in the sciatic, peroneal and tibial nerves and establish the degree and sequence of recovery after an experimental tibial shaft fracture and Ilizarov osteosynthesis.

## Materials and methods

### Experimental design

Twenty-nine adult mongrel dogs were used weighing 11–20 kg and 3–5 years of age. Experiments were carried out in accordance with the internationally agreed **Principles of Laboratory Animal Care** (**NIH** Publication **no**. **85**-**23**, revised **1985**) with the experiment protocol approved by the animal care committee at the authors’ institution. The control group consisted of five intact and nonoperated dogs, and the experimental group of 24 dogs underwent fracture and osteosynthesis.

### Modelling of fracture and surgery

**Fig. 1:**
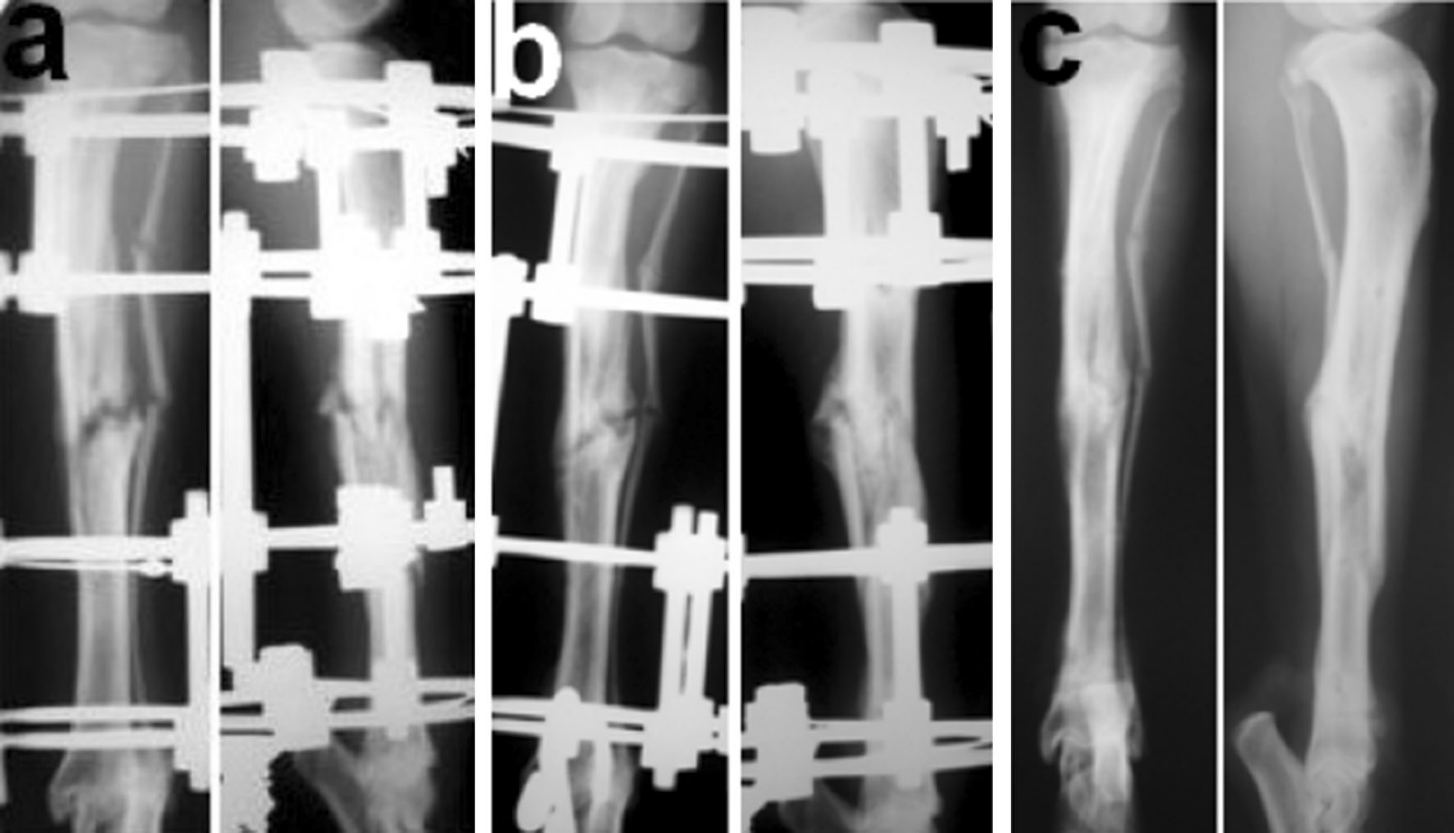
Tibial fracture in dog on standard X-ray radiographs. **a** Fixation in apparatus 21 days, **b** 50 days—the end of consolidation phase, **c** 30 days after the fixator removal

After deep intravenous combined anaesthesia and standard leg positioning, the tibial fracture was created by a standard load of 5 kg falling from the height of 1.5 m. The fracture was reduced and immobilized with a splint in the following 24 h. Ilizarov transosseous osteosynthesis was performed in aseptic conditions of operating room with a repeat of anaesthesia thereafter. Prophylactic Cefazolin was administered postoperatively for 7 days. Wounds and wire channels were monitored daily. After sedation, standard anteroposterior and lateral radiographs (Fig. 1) of the fractured tibia were made at 1-week intervals to assess fracture healing.

### Electrophysiological tests

Intramuscular EMG was performed after anaesthesia at four time-points: (1) at 37 and 50 fixation days (F37 and F50); (2) at 30 and 60–90 days postfixator removal (WA30, WA60–90). Stimulus-induced bioelectric activity (M-responses) in gastrocnemius and tibialis anterior muscles was recorded using a digital EMG-system DISA-1500 (DANTEC, Denmark). Biopotential leads were monopolar with modified needle electrodes. The active recording electrode was inserted transcutaneously in the muscle belly and the reference electrode in its tendon. M-responses were recorded after supramaximal electrical stimulation was applied to the sciatic nerve through paraneural needle electrodes using rectangular wave pulses of 1-ms duration. Muscle action potential amplitudes were measured from the top of the negative peak to the top of maximal positive peak.

### Morphological evaluation

The animals were euthanized at eight time-points: 7, 14, 20, 37, 50 fixation days (F7, F14, F20, F37, F50); also at 30, 60–90 and 120 days after the fixator removal (without apparatus WA30, WA60–90, WA120). The nerve samples were processed to obtain epoxy semithin sections at three sites: middle one-third of the thigh for sciatic nerve, middle one-third of the leg for peroneal and tibial nerves. Sections (thickness 0.5–1.0 µm) were made with glass knives using the Nova ultratome LKB (Sweden), mounted on glass slides and then stained with toluidine blue and methylene blue-basic fuchsin. The images were digitized using the photomicroscope “Opton” (Germany) connected to the “DiaMorph” software program (Russia, Moscow). Histomorphometry was performed with the “VT-Master-Morphology” program (VideoTest, Russia, St. Petersburg). In 25 nonoverlapping fields of the endoneural compartment from each nerve, collected in a systematic random order, the numerical densities of endoneural vessels (N_A_mv), myelinated and unmyelinated nerve fibres (N_A_mf and N_A_uf), and per cent of degenerated myelinated nerve fibres (Deg%) were evaluated. About 400 samples of myelinated fibres for each nerve site were made, and morphometric parameters—diameters of myelinated nerve fibres (Dmf), their axons (Dax) and myelin sheath thickness (Lmyel)—were measured.

### Statistical evaluation

The data obtained were evaluated for statistical differences using the unpaired Student *t* test, Mann–Whitney *U* test and Pearson correlation test (software package Attestat Program, version 9.3.1, developed by I. P. Gaidyshev, Certificate of Rospatent official registration No. 2002611109).

## Results

### Radiograph assessment

The 24 fractures were classified by the AO/OTA system as: four type 42A3; four type 42B1; two 42B2; nine 42B3; four 42C1; and one 42C3. Correspondingly, 16.7 % fractures were simple, 62.5 % wedge and 20.8 % complex. Using the Gustilo classification in 22 dogs, the fractures were open and were classed as types II and IIIa. The time for clinical and radiological consolidation from the date of injury to removal of Ilizarov frame varied from 42 to 50 days (46.3 ± 1.5).

### Electrophysiological tests

At the end of consolidation phase (F37 and F50), the average recorded M-response amplitudes were substantially decreased in comparison with the initial levels, especially in tibialis anterior muscles (Table 1). After fixator removal, these increased and at WA60–90 days were at 79.5 % of the initial level in gastrocnemius and 62.2 % in tibialis anterior. At the end of experiment, these parameters varied individually and did not reach initial levels.

**Table 1:** Mean (±standard deviation) of M-response amplitudes (mV) in gastrocnemius and tibialis anterior muscles before fracture, at consolidation phase (F37 and F50 days) and after the fixator removal (WA30 and WA60–90 days)

Time-points and parameter value/muscle	Ai	F37		F50		WA30		WA60–90	
		Ae	Δ%	Ae	Δ%	Ae	Δ%	Ae	Δ%
Gastrocnemius	26.8 (1.2)	8.9* (0.8)	−66.8 %	11.4* (0.7)	−57.8 %	18.2 (1.5)	−32.1 %	21.3 (2.1)	−20.5 %
Tibialis anterior	23.8 (1.3)	6.1* (1.0)	−74.4 %	6.7* (1.0)	−71.8 %	12.5* (1.3)	−47.5 %	14.8 (1.1)	−37.8 %

*Ai* initial amplitudes values (before fracture and osteosynthesis), *Ae* experimental values

Δ% = (Ai − Ae)/Ai × 100 %

* Significant differences (*t* test; *p* < 0.05) between Ai and Ae

### Microscopic observation

**Fig. 2:**
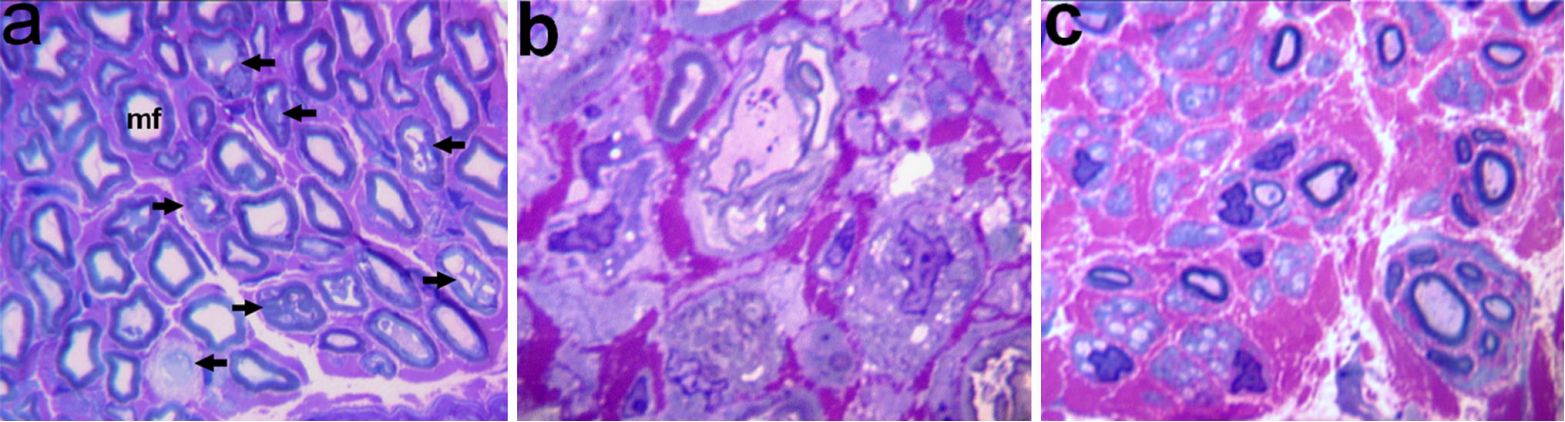
Fragments of histological transverse semithin sections of the peroneal nerve in dogs with tibial fracture at the consolidation phase. Methylene blue-basic fuchsin staining. **a**, **b** Endoneurium at 20 days of fixation in Ilizarov apparatus: *mf* normal myelinated fibre, *arrows* degenerated mf, **b** almost all myelinated and unmyelinated fibres are degenerated, **c** regenerated nerve fibres at 50 days of fixation in Ilizarov apparatus. Instrumental magnification 500× (**a**) and 1250× (**b**, **c**)

**Fig. 3:**
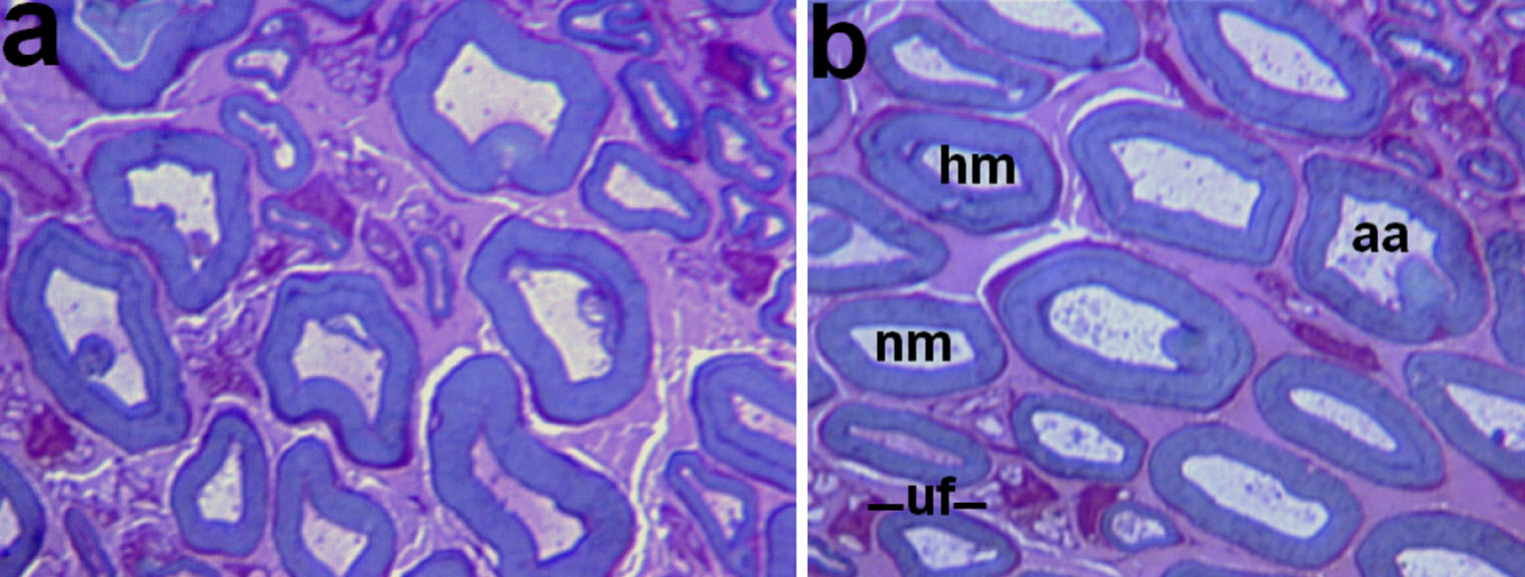
Fragments of histological transverse semithin sections of the tibial nerve in dogs with tibial fracture at 37 days of the consolidation phase. Methylene blue-basic fuchsin staining. **a** Majority of nerve fibres with myelin decompaction and axonal atrophy, **b** various nerve fibres: *nm* normally myelinated, *hm* hypermyelinated, *aa* severe axonal atrophy, *uf* profiles of unmyelinated nerve fibres containing nuclei. Instrumental magnification 1250×

After 7, 14 and 20 fixation days, the peroneal nerve epineurium was oedematous with loci of haemorrhage and paravasal lymphocytic or plasmocytic infiltration. A high content of macrophages, mast cells and plasmocytes was noted in the epineurium until 50–80 days of the experiment. Many of epineural blood vessels possessed thickened walls and widened lumens, with signs of myocyte dystrophy and death. Large arterioles and capillaries with widened lumens were seen in the endoneural vessels which is unusual for intact nerves. In some fascicles, signs of perineuritis were noted. Axonal and Wallerian degeneration or demyelination occurred more often in thick myelinated fibres at early time-points (F7–F20) in peroneal nerve (Fig. 2a, b), but were very rare in the tibial nerves. Unmyelinated fibre loss was seen in the peroneal nerve (Fig. 2b). At F50 nerve fibre regeneration was the dominant picture. Some sections of regenerated fibres contained elements of paranodal sprouting and were encircled with lemmocytic proliferates; these are signs of repeating de- and remyelination (Fig. 2c). Nerve fibre hypermyelination was typical in the tibial nerve from F20 until the end of experiment (Fig. 3a, b). Most of the unmyelinated nerve fibres contained few axons but many Schwann cell nuclei. An increased number of Remak bundles containing small myelinated axons occurred in tibial nerve, much more than would be seen for an intact nerve. The same remodelling of Remak bundles was noted in sciatic nerve.

### Morphometric findings

Table 2 shows that in the leg (tibial and peroneal nerves), maximal degeneration was seen at F7, whereas in the sciatic nerve this occurred at F14.

**Table 2:** Per cent rates—mean (±standard deviation)—of degenerated myelin fibres (Deg%) in sciatic, peroneal and tibial nerves of intact and operated animals

Group and time-point/nerve	Intact animals	Operated animals							
		F7	F14	F20	F37	F50	WA30	WA60–90	WA120
Sciatic	1.79 (0.31)	1.85 (0.14)	6.13 (2.34)	5.12 (2.23)	4.25 (1.89)	3.11 (1.68)	3.44 (1.42)	2.89 (1.43)	4.12 (1.09)
Peroneal	1.92 (0.31)	12.51 (2.09)	11.79 (0.30)	8.43 (2.24)	4.64 (0.85)	5.66 (3.52)	5.00 (0.31)	4.74 (0.35)	4.16 (1.00)
Tibial	1.64 (0.20)	10.11 (0.20)	8.65 (3.47)	4.82 (2.32)	5.81 (1.30)	5.44 (0.80)	4.82 (0.37)	5.00 (0.96)	3.77 (0.53)

**Fig. 4:**
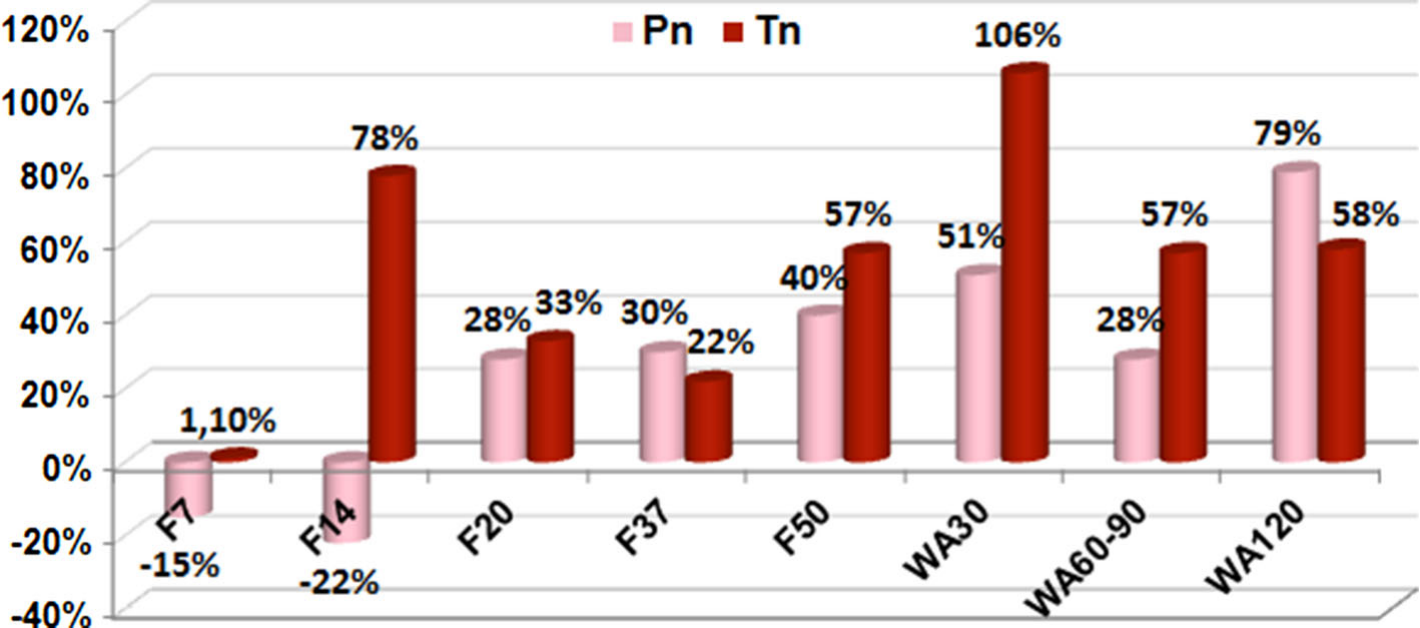
Endoneural vessel quantification. Per cents of differences of endoneural vessel densities between nerves of intact (i) and experimental (e) animals (N_A_mv-i − N_A_mv-e/N_A_mv-i × 100 %) in peroneal (Pn) and tibial (Tn) nerves at various time-points of experiment (F7–F50—days of consolidation phase; WA30–WA120—days after the fixator removal)

Figure 4 shows the differences of endoneural vessel densities between nerves of experimental and intact animals. In the peroneal nerve after the first 2 weeks of experiment, endoneural hypovascularity was noted. From F20 until the end of the experiment, the endoneurium of the peroneal nerve of experimental animals was more vascularized than that of intact group. In tibial nerve at F7, N_A_mv did not change from those in the intact group, but endoneural hypervascularity was recorded with maximums at F14 and WA30 (Fig. 4).

**Fig. 5:**
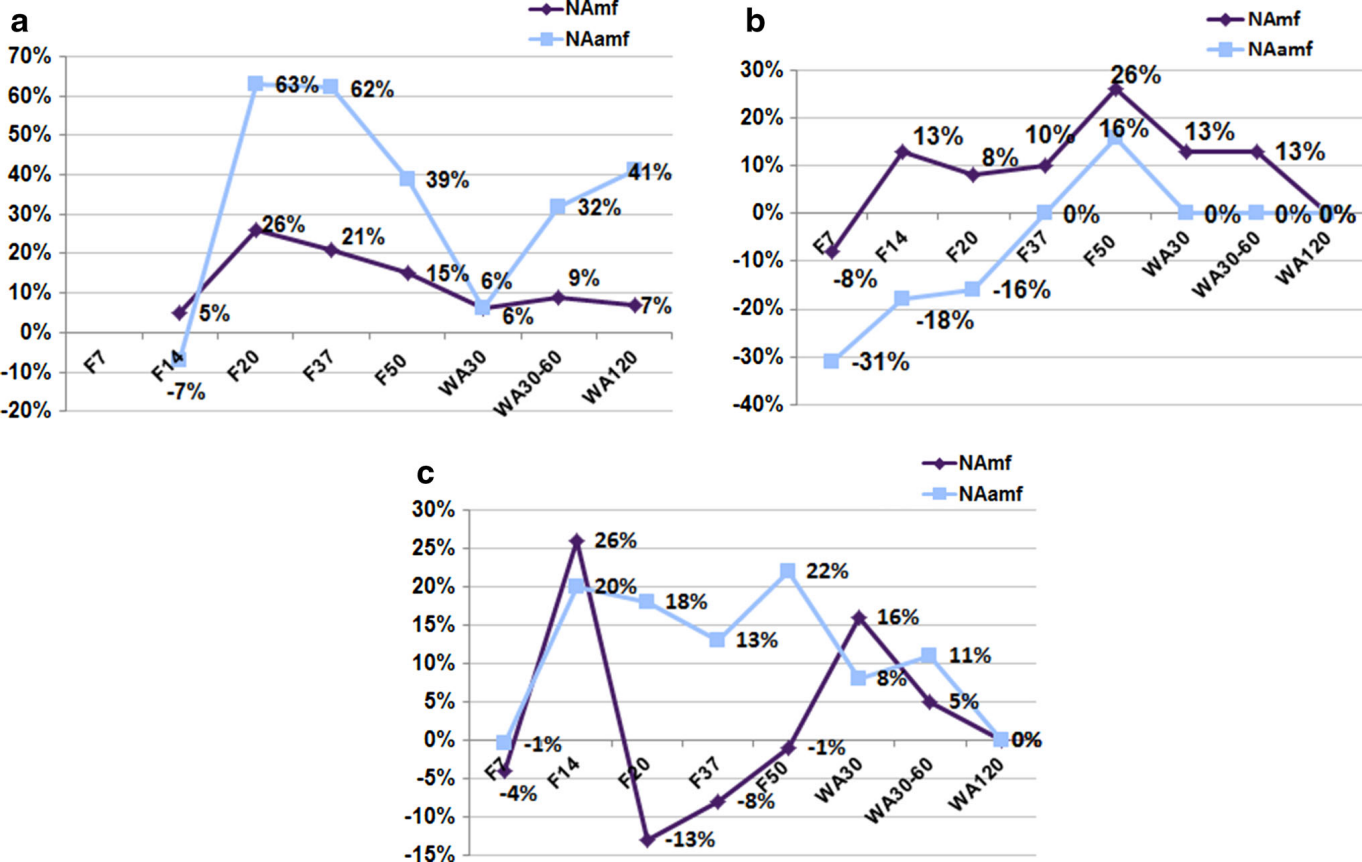
Numerical densities of myelinated and unmyelinated nerve fibres (N_A_mf and N_A_uf). Per cents of differences of nerve fibre densities between nerves of intact and experimental animals at various time-points of experiment (F7–F50—days of consolidation phase; WA30–WA120—days after the fixator removal) **a** sciatic nerve, **b** peroneal nerve, **c** tibial nerve

Figure 5 demonstrates the differences of nerve fibre densities between nerves of experimental and intact animals. In the sciatic nerve, the parameters differed significantly from the intact group at all time-points. At F14 the numerical density of unmyelinated fibres was slightly lower but that of myelinated fibres slightly higher than in intact nerves, but both then increased to 26 and 63 %, respectively. From F14 to WA30, these decreased and then increased again until the end of the experiment. The oscillation of unmyelinated nerve fibre densities was more marked. In the leg the tibial and peroneal nerves had, at the beginning of experiment, nerve fibre densities lower than in intact nerves especially with the unmyelinated nerve fibre density in the peroneal nerve (Fig. 5). In the peroneal but not in the tibial nerve, oscillations of myelinated and unmyelinated nerve fibre densities were synchronized. At the end of experiment, numerical nerve fibre densities of experimental animals did not differ from nerves in the intact group.

A notable correlation was found between numerical densities of endoneural vessels and unmyelinated nerve fibres in the peroneal nerve (*r* = 0.71) and between numerical densities of endoneural vessels and myelinated nerve fibres in the tibial nerve (*r* = 0.66).

Table 3 shows changes in size of myelinated nerve fibres and their axons in peroneal and tibial nerves of experimental animals in comparison with the intact group. At F7, the average diameters of myelinated nerve fibres and their axons were lower than in intact nerves, but, in the tibial nerves, myelin sheaths were thicker on average than in intact nerves. The greatest decrease in average diameters of myelin fibres and theirs axons was noted at F50 and WA30. Myelin sheaths of nerve fibres in peroneal nerves at F14 and the following time-points of the consolidation phase were significantly thinner on average than in intact nerves especially at F37, but, after fixator removal, the parameter recovered. For nerve fibres of the tibial nerve, a sustained increase in myelin thickness was seen.

**Table 3:** Mean (±standard deviation) of morphometric parameters (mkm) of myelinated fibres (MF) in peroneal and tibial nerves of intact (I) animals and in nerves of operated animals

Group and time-point/nerve parameter	I	Operated animals							
		F7	F14	F20	F37	F50	WA30	WA60–90	WA120
Peroneal									
MF diameter	6.46 (0.07)	6.29* (0.70)	6.08* (0.08)	5.98* (0.32)	5.78* (0.15)	5.03* (0.56)	5.67* (0.20)	5.89* (0.07)	6.21* (0.39)
Axonal diameter	4.39 (0.08)	4.18* (0.53)	4.17* (0.01)	4.04* (0.35)	3.73* (0.19)	3.40* (0.20)	3.42* (0.06)	3.98* (0.14)	4.15* (0.24)
Myelin thickness	1.04 (0.04)	1.06 (0.11)	0.95* (0.04)	0.97* (0.01)	0.67* (0.48)	0.82* (0.13)	1.00* (0.07)	0.96* (0.05)	1.07 (0.09)
Tibial									
MF diameter	6.75 (0.28)	7.20* (2.91)	6.01* (0.07)	6.44* (0.35)	6.58* (0.35)	6.28* (0.64)	6.31* (0.41)	6.46* (0.36)	6.76 (0.10)
Axonal diameter	4.63 (0.33)	4.18* (1.62)	3.95* (0.44)	4.00* (0.33)	4.14* (0.37)	3.80* (0.21)	3.56* (0.05)	3.97* (0.15)	4.25* (0.16)
Myelin thickness	1.06 (0.05)	1.51* (0.74)	1.03 (0.19)	1.22* (0.17)	1.22* (0.03)	1.24* (0.24)	1.24* (0.23)	1.22* (0.24)	1.25* (0.03)

* Significant differences (Mann–Whitney *U* test; *p* < 0.01) between experimental and intact groups

A strong correlation was established between the mid-values of the amplitudes of M-responses in tibialis anterior and the average nerve fibre size of the peroneal nerve: *r* values for relation between the M-responses and the average myelin nerve fibre diameter, axonal diameter and myelin thickness were 0.84, 0.92 and 0.65, respectively. As for the tibial nerve, a positive correlation between the mid-values of the amplitudes of M-responses in gastrocnemius was established with the average myelin nerve fibre diameter and axonal diameter (*r* values 0.69 and 0.57, respectively). For an average myelin thickness *r* = −0.91, indicates a negative functional significance of hypermyelination.

## Discussion

The recovery of contractile muscle action after tibial fractures is problematic especially for sportsmen [18]. To assess a neurologic impact in such a condition, we have studied histological changes in the peroneal, tibial and sciatic canine nerves after experimental leg fractures. Severe nerve injuries arising from bone fragments were not shown. We used an Ilizarov fixator for fracture stabilization, but nerve injuries from the wires were also not shown. Bone union was achieved earlier than after hybrid fixator use in dogs [19].

A greater deficit of bioelectric activity was seen in tibialis anterior muscle than in gastrocnemius. Substantial differences in histological changes within the peroneal and tibial nerves were also recorded. In the peroneal nerve, we observed more destructive changes in the epineurium, endoneural hypovascularity and striking loss of unmyelinated nerve fibres. In spite of active neuroregeneration and remyelination at the end of consolidation phase, even at 120 days after fixator removal, axonal atrophy was sustained and the percentage of degenerated myelinated fibres was higher than in intact nerves. The M-responses amplitudes in tibialis anterior muscle was 40 % lower than initial levels. In tibial nerve, in spite of better preservation of unmyelinated nerve fibres, axonal atrophy was also seen together with degenerative hypermyelination. A probable explanation of differences between peroneal and tibial nerves changes is the diversity of microvascular compression, confirmed by dynamics of endoneural vascularity and consistent with other authors’ clinical data about pressure in closed fascial leg compartments after isolated tibial fractures [20]. It is known that in patients with trigeminal neuralgia, the various types of demyelination correspond to the extent of trigeminal root compression [21].

An unexpected finding in our research was a substantial increase in nerve fibre numerical densities at some time-points of experiment due to remodelling of Remak bundles. The first peak was at 14 days of fixation and the second at the end of consolidation phase. An increase in nerve fibre numerical densities and myelination of unmyelinated axons in Remak bundles was observed in the sciatic nerve of the rat after intraperitoneal injections of GDNF [22]. While the inductive effect of GDNF on osteoblasts proliferation is well known [23], expression of various neurotrophins and their receptors by osteogenic cells has also been shown [24]. It is possible that the fluctuations of numerical nerve fibre densities in the sciatic, peroneal and tibial nerves after tibial fractures reflect a regulatory role of the nervous system in fracture healing.

A limitation of this study is the absence of data on neural growth factor expression. Further research is needed about the relation and influence of the identified destructive and adaptive nerve changes on fracture healing.

## Conclusion

In this animal study, we show, for the first time, that even in the absence of nerve injuries either from the fracture or through the application of an Ilizarov fixator, partial degenerative changes, axonal atrophy and demyelination of nerve fibres occur in the peroneal and tibial nerves which influence muscle bioelectric activity negatively during the period of fracture healing and for a long time after fixator removal.
